# Deficiency of TET2-mediated KMT2D self-transcription confers a targetable vulnerability in hepatocellular carcinoma

**DOI:** 10.1093/pnasnexus/pgae504

**Published:** 2024-11-11

**Authors:** Yuting Jin, Keqiang Rao, Jiaojiao Zheng, Xinchao Zhang, Yi Luo, Jing He

**Affiliations:** Department of Liver Surgery, Renji Hospital, Shanghai Jiao Tong University School of Medicine, Shanghai 200120, China; Department of Liver Surgery, Renji Hospital, Shanghai Jiao Tong University School of Medicine, Shanghai 200120, China; Department of General Surgery, Zhongshan Hospital, Fudan University, Shanghai 200032, China; Department of Pathology, College of Basic Medical Sciences, Shanghai Jiao Tong University School of Medicine, Shanghai 200025, China; Department of Liver Surgery, Renji Hospital, Shanghai Jiao Tong University School of Medicine, Shanghai 200120, China; Department of Liver Surgery, Renji Hospital, Shanghai Jiao Tong University School of Medicine, Shanghai 200120, China

**Keywords:** TET2, KMT2D, self-transcription

## Abstract

Hepatocellular carcinoma (HCC) has become a leading cause of cancer-related mortality worldwide. Conventional therapies tend to exacerbate comorbidities, liver dysfunction, and relapse, rendering an urgent demand for novel strategy for management of HCC. Here, we reported that DNA dioxygenase TET2 collaborates with histone methyltransferase KMT2D to enable transcription of *KMT2D* and *ARID1A* in HCC. Mechanistically, *KMT2D* and *ARID1A* are the major epigenetic targets of TET2 through RNA-seq analysis. Moreover, KMT2D recruits TET2 to facilitate self-transcription via oxidation of 5-methylcytosine in promoter, thereby maintaining expression of ARID1A. Physiologically, KMT2D was identified as a tumor suppressor and mediates the antitumor effect of vitamin C in HCC. Tumors with depleted KMT2D present growth advantage over control group. Vitamin C is able to impair tumor growth, which is compromised by deficiency of KMT2D. Furthermore, loss of KMT2D sensitizes HCC tumors to cisplatin with reduced tumor weight and high level of DNA damage. Ultimately, TET2–KMT2D axis correlates with prognosis of patients with HCC. Patients with high amounts of TET2 and KMT2D present better outcome. Our findings not only put forth a heretofore unrecognized mechanism underlying cross-talk between TET2 and KMT2D in mediating self-transcription of KMT2D, but also propose a targetable vulnerability for HCC therapy on the basis of TET2–KMT2D axis.

**Significance Statement**

Hepatocellular carcinoma has become a leading cause of cancer-related mortality worldwide, highlighting the urgent demands for novel therapeutic strategy to alleviate the burden of HCC worldwide. In this study, we found that histone methyltransferase KMT2D recruits TET2 to facilitate self-transcription and expression of ARID1A via oxidation of 5-methylcytosine in promoters. Furthermore, we revealed that loss of KMT2D sensitizes HCC tumors to cisplatin. TET2–KMT2D axis correlates with prognosis of patients with HCC.

## Introduction

Hepatocellular carcinoma (HCC), the most common subtype of primary liver malignancy, has become a leading cause of cancer-related mortality worldwide (1). Conventional managements of HCC involve surgical resection, transplantation, ablation, transarterial embolization and radiotherapy, and diverse systemic therapies, whereas most of therapies tend to exacerbate patient comorbidities and liver dysfunction (2), implying that a comprehensive decision-making process should be taken into consideration. Therefore, HCC surveillance programs improvement and novel therapeutic strategy development are urgent demands to prolong lifespan of patients and alleviate the burden of HCC worldwide till yet.

Genomic alteration and epigenetic modification are frequently accumulated accompanied with onset of HCC (3), which underlines the clinical relevance between epigenetic marks and HCC progression. 5-Methylcytosine (5mC), accounting for 60–80% of CpG sites in mammals, is the major form of DNA modification and plays a critical role in development and disease (4). 5-Hydroxymethylcytosine (5hmC), the oxidation state of 5mC, impacts a broad range of physiological processes across tumors. Intriguingly, 5hmC and DNA dioxygenase TET2 are indeed epigenetic signatures linked to HCC progression (5). TET family proteins function as DNA dioxygenase to regulate gene expression via catalyzing 5mC into 5hmC, 5-formylcytosine, and 5-carboxylcytosine iteratively (6). Unlike TET1 and TET3, TET2 is frequently mutated and characterized by loss of function in myeloid cancers (7, 8), implying the critical role of TET2 across cancers. Loss of TET2 initiates aberrant self-renewal of hematopoietic stem cells and onset of myeloid malignancies (9, 10). Subsequently, moonlighting functions of TET2 were gradually uncovered such as oxidation of mRNA or tRNA (11, 12) and recruiting Hdac2 to repress gene transcription (13).

A plethora of evidences have proposed a mutually exclusive interplay between DNA methylation and H3K4 methylation, noting that H3K4 methylation, written by KMT family, is vital for gene transcription (14, 15). KMT2A lacking CXXC domain results in increased DNA methylation at CpG islands (16). Consistently, unmethylated CpG islands assure KMT2F and KMT2G localized to promoter (17). The integrity of H3K4 methyltransferase SET1/COMPASS complex is improved via GlcNAcylation of its component HCF1 triggered by interaction between TET2/3 and OGT (18). These results underscore the interplay between DNA methylation or TET proteins with histone methylation, suggesting that communications within epigenetic enzymes may play critical roles in coordinating complex cellular processes. Nevertheless, direct cross-talk among TET2 and other epigenetic modulators remains to be elucidated.

Here, we reported that KMT2D and ARID1A are major downstream targets of TET2 among various epigenetic enzymes in HCC cells. Mechanistically, TET2 is recruited by KMT2D to facilitate self-transcription via oxidation 5mC in promoter. TET2 is required for expression of ARID1A induced by KMT2D. Moreover, KMT2D functions as a tumor suppressor and mediates a general antitumor effect of TET2 agonist vitamin C in HCC. Deficiency of KMT2D sensitizes HCC tumors to cisplatin. Eventually, we found that TET2–KMT2D axis correlates with prognosis of HCC. This study not only proposes the direct evidence of interaction between epigenetic modulators, but also provides a targetable vulnerability for HCC lacking KMT2D.

## Results

### TET2 facilitates transcription of *KMT2D* and *ARID1A* in HCC cells

**Fig. 1.**
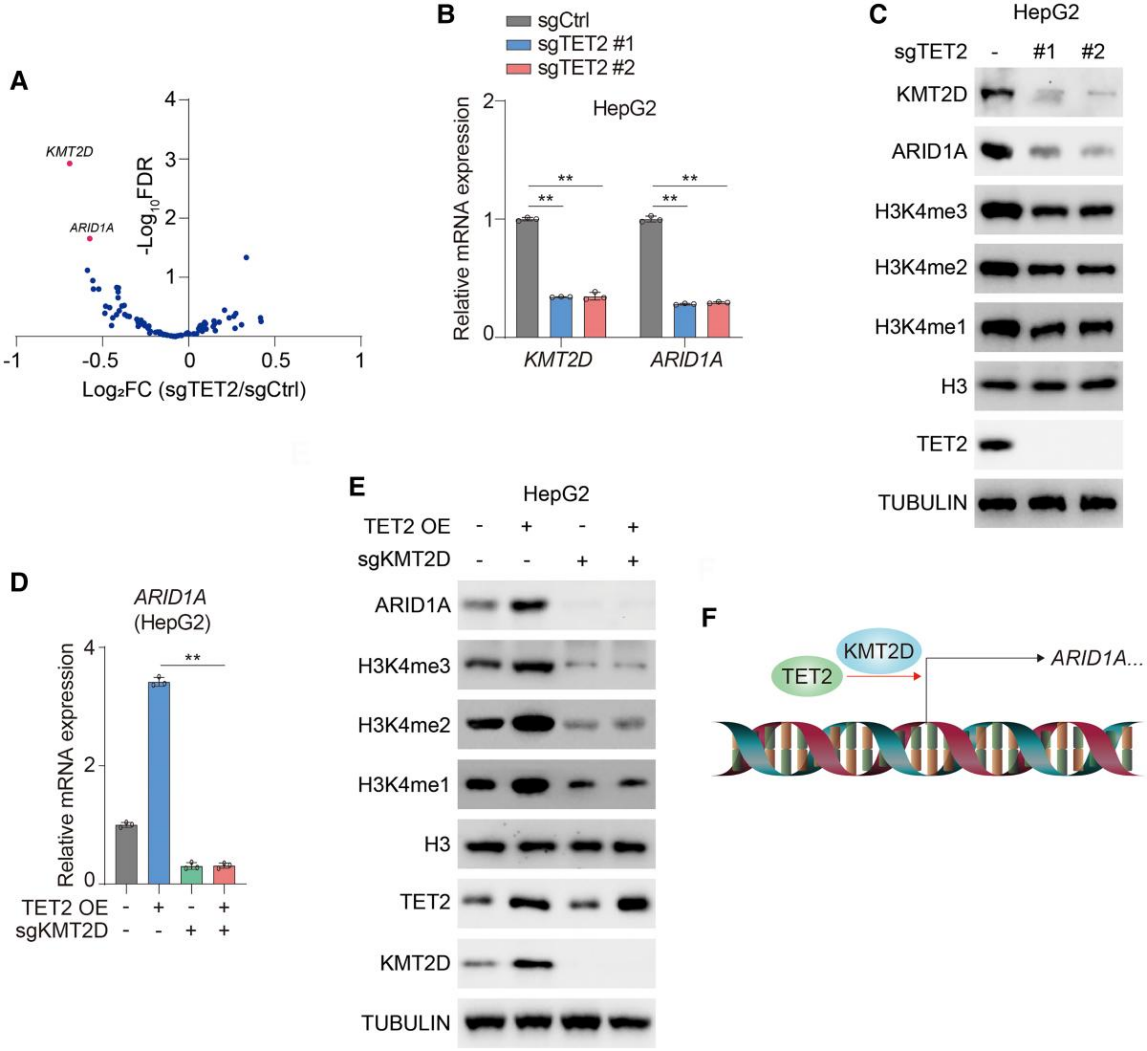
TET2 promotes expression of *KMT2D* and *ARID1A* in HCC cells. A) Expression of *KMT2D* and *ARID1A* is significantly changed among various epigenetic modulators in sgTET2 cells through RNA-seq analysis. Volcano plot showing RNA profiling for HepG2 cells deletion of TET2 compared with control cells. B, C) HepG2 cells were transfected with or without sgRNAs targeting TET2 (sgTET2). mRNA (B) and protein (C) levels of KMT2D and ARID1A were analyzed. Immunoblotting analysis was performed using the indicated antibodies (C). D, E) HepG2 cells were transfected with or without sgRNAs targeting KMT2D (sgKMT2D) and overexpressed with or without TET2. mRNA (D) and protein (E) levels of ARID1A were analyzed. Immunoblotting analysis was performed using the indicated antibodies (E). F) Schematic of transcription of *KMT2D* and *ARID1A* initiated by TET2. Data are presented as mean ± SD, *n* = 3 independent repeats. Unpaired, two-tailed t test; **P < 0.01.

To determine potential cross-talk among TET2 and other epigenetic modulators, we examined the RNA-seq results of TET2 knockout HCC cells and found that among diverse epigenetic factors, *KMT2D* and *ARID1A* are strikingly decreased upon deletion of TET2 (Fig. 1A). KMT2D is a histone methyltransferase and regulates chromatin structure by orchestrating H3K4 methylation, thereby enhancing gene expression and maintaining cellular development and differentiation (19). ARID1A is a core component of SWI/SNF chromatin-remodeling complex and contributes to tumor suppression (20). ARID1A is reported as a potential target of KMT2D (21). Consistent with RNA-seq results, deficiency of TET2 remarkably impairs expression of KMT2D and ARID1A (Figs. 1B and C, S1A and B). To verify whether KMT2D is required for expression of ARID1A, we introduced TET2 into KTM2D knockout cells. As a consequence, knockout of KMT2D notably impairs transcription of ARID1A (Figs. 1D and E, S1C and D). In contrast, overexpression of TET2 significantly boosts ARID1A transcription, which is compromised by deletion of KMT2D (Figs. 1D and E, S1C and D), suggesting that TET2 enables transcription of ARID1A via forcing expression of KMT2D. To strengthen these findings, we conducted ARID1A knockout cells with overexpression of TET2 as well. Deficiency of ARID1A exerts a minor effect on expression of KMT2D (Fig. S1E and F). Moreover, TET2 regulates expression of KMT2D independent of ARID1A (Fig. S1E and F). Taken together, TET2 enhances transcription of *KMT2D*, thereby supporting expression of ARID1A in HCC (Fig. 1F).

### TET2 oxidizes 5mC to enhance transcription of *KMT2D* and *ARID1A*

**Fig. 2.**
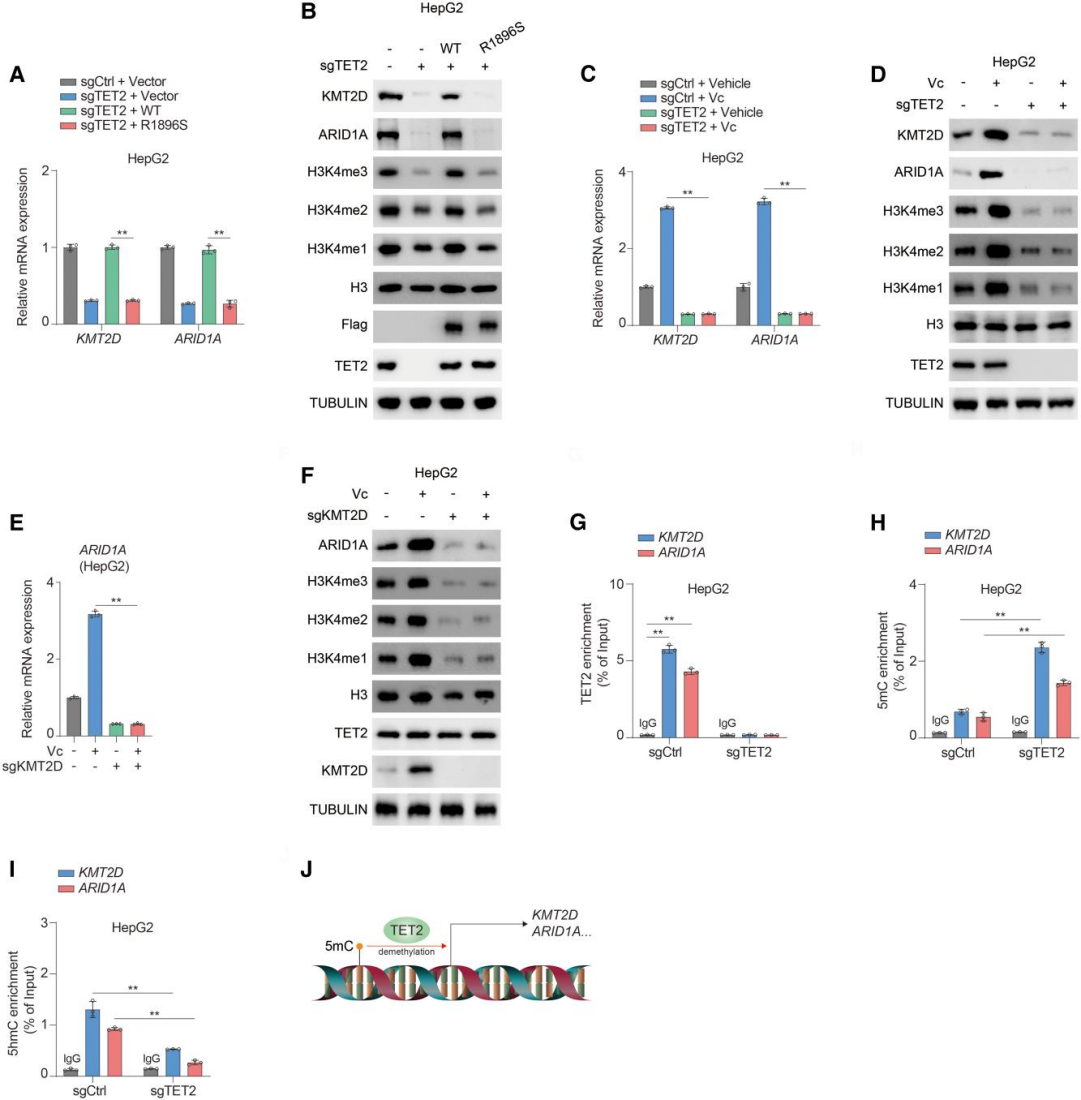
TET2 initiates transcription of *KMT2D* and *ARID1A* via oxidizing 5mC of promoters. A, B) sgCtrl and sgTET2 HepG2 cells were transfected with or without wild-type TET2 (WT) and TET2 catalytic mutant (R1896S). mRNA (A) and protein (B) levels of KMT2D and ARID1A were analyzed. Immunoblotting analysis was performed using the indicated antibodies (B). C, D) sgCtrl and sgTET2 HepG2 cells were treated with or without 1 mM Vc for 24 h. mRNA (C) and protein (D) levels of KMT2D and ARID1A were analyzed. Immunoblotting analysis was performed using the indicated antibodies (D). E, F) sgCtrl and sgKMT2D HepG2 cells were treated with or without 1 mM Vc for 24 h. mRNA (E) and protein (F) levels of ARID1A were analyzed. Immunoblotting analysis was performed using the indicated antibodies (F). G–I) ChIP assay was performed in sgCtrl and sgTET2 HepG2 cells using antibodies against TET2 (G), 5mC (H), and 5hmC (I). DNA enrichment was examined by quantitative real-time PCR. The *y* axis shows the value normalized to input. J) Schematic of TET2-mediated transcription of *KMT2D* and *ARID1A* by oxidation of 5mC in promoters. Data are presented as mean ± SD, *n* = 3 independent repeats. Unpaired, two-tailed t test; **P < 0.01.

Given that TET2 functions as DNA dioxygenase to maintain transcription of genes (22), mutation of Arg1896 in TET2 to Ser (R1896S) was created as previously reported (11) to explore the catalytic effect of TET2 on KMT2D and ARID1A. We re-introduced wild-type (WT) TET2 and its catalytic mutant R1896S into TET2 knockout cells and found that compared with R1896S, WT TET2 is sufficient to rescue global 5hmC levels (Fig. S2A) and repression of KMT2D and ARID1A induced by deletion of TET2 (Figs. 2A and B, S2B and C). As vitamin C was demonstrated as a potent agonist of TET2 activity (9), we employed vitamin C to examine catalytic regulation of TET2 on KMT2D and ARID1A in depth. As expected, vitamin C robustly induces expression of KMT2D and ARID1A, which is impaired by deficiency of TET2 (Figs. 2C and D, S2D and E). Likewise, depletion of KMT2D diminishes the elevation of ARID1A induced by vitamin C (Figs. 2E and F, S2F and G), underlying the vital role of KMT2D in mediating expression of ARID1A initiated by TET2. To reinforce these findings, we performed chromatin immunoprecipitation (ChIP) assay and found that TET2 associates with promoters of *KMT2D* and *ARID1A* (Figs. 2G and S2H), which was validated by knockdown (Fig. S2I and J) and overexpression (Fig. S2K and L) of TET2. Furthermore, deficiency of TET2 dramatically increases 5mC level of *KMT2D* and *ARID1A* promoters (Figs. 2H and S2M), whereas 5hmC levels of *KMT2D* and *ARID1A* promoters are significantly reduced upon depletion of TET2 (Figs. 2I and S2N). To fully explore methylation levels of *KMT2D* and *ARID1A* promoters, we conducted whole-genome bisulfite sequencing analysis and found that methylation level of *KMT2D* promoter is significantly induced by loss of TET2 (Fig. S2O). Unfortunately, the methylation level of *ARID1A* promoter remains unchanged upon deletion of TET2 (Fig. S2O). Nevertheless, loss of TET2 leads to increased methylation sites within *ARID1A* promoter (Fig. S2O), suggesting that TET2 exerts a potential effect on methylation regulation of *ARID1A* promoter. Collectively, TET2 promotes transcription of *KMT2D* and *ARID1A* via oxidation of 5mC in promoters (Fig. 2J).

### KMT2D recruits TET2 to support self-transcription

**Fig. 3.**
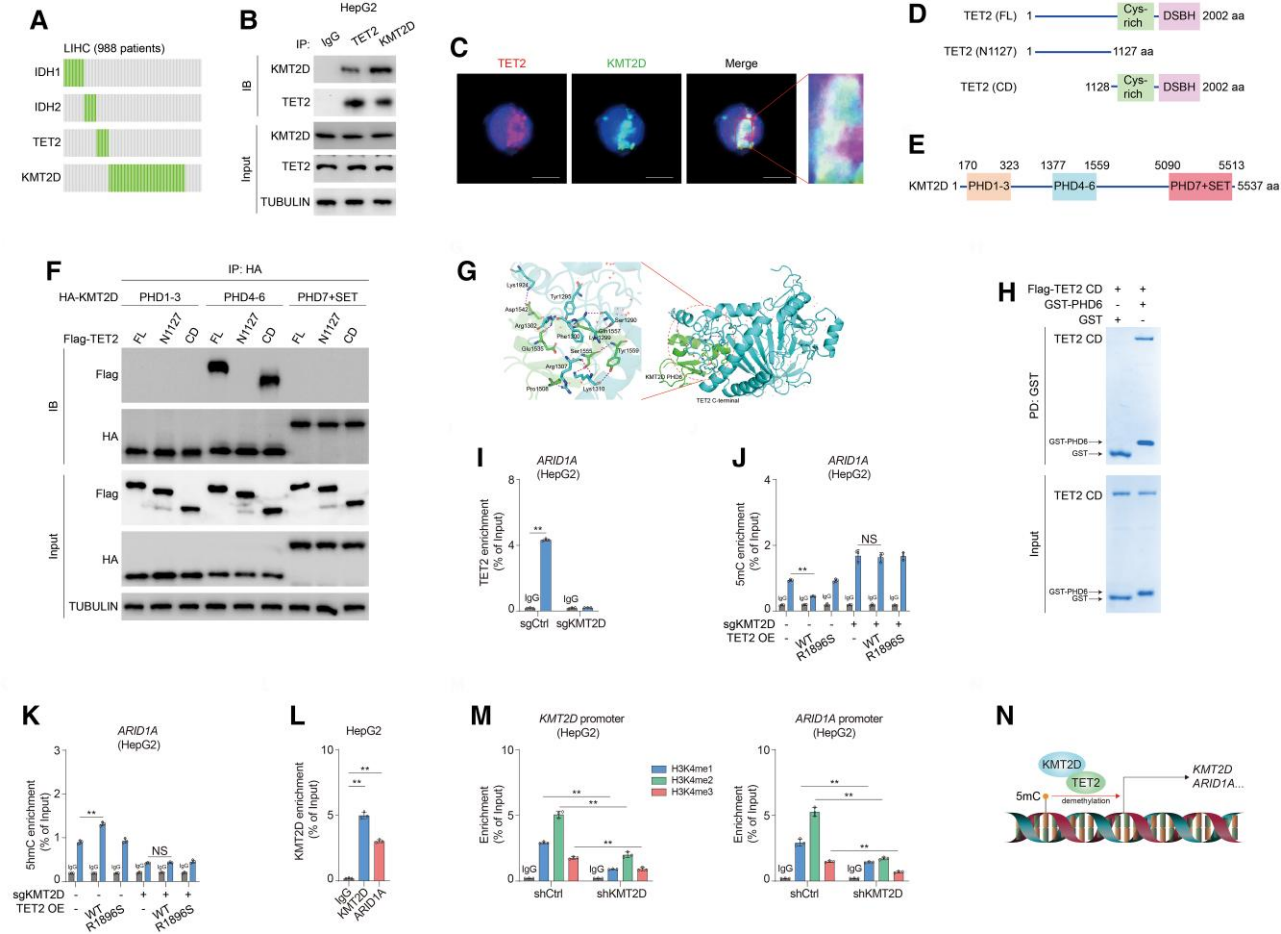
TET2 is recruited by KMT2D to facilitate self-transcription. A) Mutations in IDH1/2–TET2 axis and KMT2D display mutual exclusivity in LIHC. B, C) TET2 interacts with KMT2D. Whole cellular extracts were subjected to IP using the indicated antibodies in HepG2 cells (B). Immunofluorescence analysis using indicated antibodies was performed (C). D) Structural description of TET2-truncated mutants. E) Structural description of KMT2D domains. F) 239T cells stably expressing HA-tagged indicated KMT2D domains were transfected with indicated TET2-truncated mutants. Whole cellular extracts were subjected to IP using beads conjugated with antibody against HA. Immunoblotting analysis was performed using the indicated antibodies. G) Docking analysis of PHD6 domain of KMT2D (PDB accession code: 8U2Y) and C terminus of TET2 (PDB accession code: 4NM6). H) PD assay of TET2 CD and KMT2D PHD6. TET2 CD was purified from 293T cells stably expressing Flag-tagged TET2 CD. KMT2D PHD6 was purified from *E. coli* expressing GST-tagged PHD6. PD. I) ChIP assay was performed in sgCtrl and sgKMT2D HepG2 cells using antibodies against TET2. DNA enrichment was examined by quantitative real-time PCR. The *y* axis shows the value normalized to input. ChIP assay was performed in sgCtrl and sgKMT2D HepG2 cells transfected with or without WT and R1896S TET2 using antibodies against 5mC (J) and 5hmC (K). DNA enrichment was examined by quantitative real-time PCR. The *y* axis shows the value normalized to input. L) ChIP assay was performed in HepG2 cells using antibodies against KMT2D. DNA enrichment was examined by quantitative real-time PCR. The *y* axis shows the value normalized to input. M) ChIP assay was performed in HepG2 cells using antibodies against H3K4me1, H3K4me2, and H3K4me3. DNA enrichment was examined by quantitative real-time PCR. The *y* axis shows the value normalized to input. N) Schematic of self-transcription of *KMT2D* by recruiting TET2 to its promoter. Data are presented as mean ± SD, *n* = 3 independent repeats. Unpaired, two-tailed t test; **P < 0.01. NS, not significant.

In regard to previous studies that mutation of IDH1/2 or TET2 always confers loss of function of TET2 (8, 23), we found that IDH1/2 and TET2 are mutually exclusive with KMT2D through TCGA database (Figs. 3A and S3A), implying that TET2 may collaborate with KMT2D to maintain gene expression. Immunoprecipitation (IP) and immunofluorescence analysis revealed that TET2 binds to KMT2D in nucleus indeed (Figs. 3B, C and S3B). To explore the interaction in depth, we constructed truncated mutants of TET2 and KMT2D (Fig. 3D and E). The results showed that TET2 catalytic domain (CD) associates with PHD4-6 domain of KMT2D (Fig. 3F). Docking analysis revealed that PHD6 domain of KMT2D directly binds to C terminus of TET2 (Fig. 3G), which is validated by pull-down (PD) assay (Fig. 3H), implying that TET2 directly contacts with KMT2D via CD–PHD6 interaction. Furthermore, deletion of KMT2D distinctly abrogates association between TET2 and *ARID1A* promoter (Figs. 3I and S3C). Next, WT and R1896S TET2 were introduced into KMT2D-deficent cells (Fig. S3D). WT TET2, rather than R1896S, recovers the declined 5hmC level caused by loss of KMT2D (Fig. S3E). Consistently, lack of KMT2D enhances 5mC but disrupts 5hmC level of *ARID1A* promoter (Figs. 3J and K, S3F and G), whereas neither WT nor R1896S TET2 enables recovery oxidation of 5mC (Figs. 3J and K, S3F and G), implying that KMT2D is required for TET2-mediated transition of 5mC to 5hmC. Given transcription factors such as WT1 and SNIP1 are critical for TET2-mediated oxidation of target gene promoter methylation (24, 25), our findings provoke the hypothesis that TET2 is recruited by KMT2D to enable self-transcription and other target genes as well. To verify the hypothesis, ChIP assay of KMT2D was conducted in HCC cells. As a result, KMT2D is unraveled bound to promoters of *KMT2D* and *ARID1A* (Figs. 3L and S3H), rendering the evidence that KMT2D recruits TET2 to facilitate self-transcription. To enhance the evidence, we performed knockdown of KMT2D (Fig. S3I), which leads to reduced expression of ARID1A and methylation of H3K4 (Fig. S3I and J). Moreover, knockdown of KMT2D strikingly dampens methylation enrichment of H3K4 in *KMT2D* and *ARID1A* promoters (Figs. 3M, S3K and L). Furthermore, we analyzed ChIPseq data of NCBI database (accession number GSE36620) and found that knockdown or knockout of TET2 impairs H3K4me3 enrichment of *KMT2D* and *ARID1A* genes including regions around promoters (Fig. S3M and N). Together, our findings demonstrated that KMT2D recruits TET2 to support self-transcription via oxidation of 5mC in promoter (Fig. 3N).

### KMT2D mediates antitumor effect of vitamin C

**Fig. 4.**
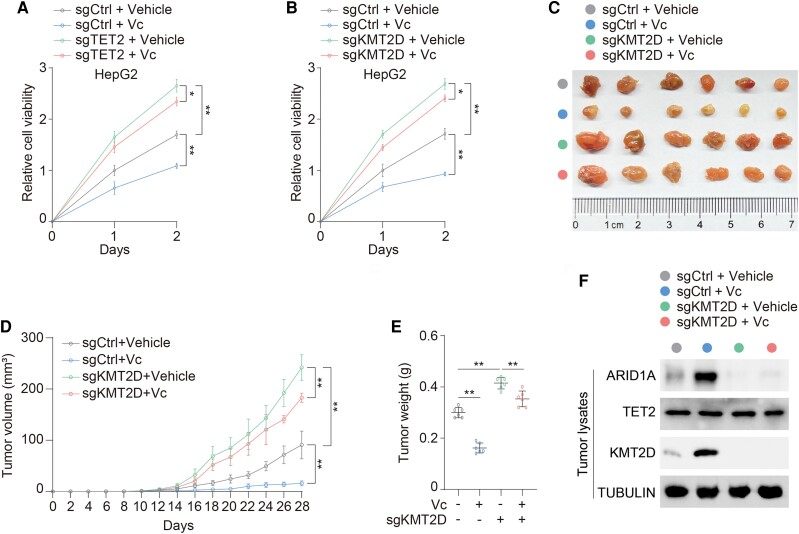
KMT2D is crucial for antitumor effect of vitamin C in HCC. A) Cell viability was analyzed in sgCtrl and sgTET2 HepG2 cells treated with or without 1 mM Vc for the indicated days. B) Cell viability was analyzed in sgCtrl and sgKMT2D HepG2 cells treated with or without 1 mM Vc for the indicated days. C–F) sgCtrl and sgKMT2D MHCC97H cells were subcutaneously injected into athymic nude mice administrated with or without Vc. Representative tumor xenografts (C). Mice were sacrificed in week 4, and tumor volume (D) and weight (E) were calculated. *n* = 6 independent animals. Tumor lysates harvested were subjected to immunoblotting analysis as indicated. Immunoblotting analysis was performed using the indicated antibodies (F). Data are presented as mean ± SD, *n* = 3 independent repeats. Unpaired, two-tailed t test; *P < 0.05, **P < 0.01.

To decipher the physiological relevance of our findings, vitamin C was employed for management of HCC. Consistent with the previous study (26), loss of TET2 compromises the antitumor effect of vitamin C in HCC (Figs. 4A and S4A). In regard to the physiological role of KMT2D in HCC, deficiency of KMT2D substantially improves cell viability of HCC cell, providing the evidence that KMT2D exerts a tumor-suppressive role in HCC cells. Moreover, deletion of KMT2D, mimicking loss of TET2, narrows down a suppressive effect of vitamin C (Figs. 4B and S4B). To define the in vivo behavior of KMT2D in HCC, we subcutaneously injected control and KMT2D knockout HCC cells into athymic nude mice. Loss of KMT2D greatly promotes tumor growth (Fig. 4C and D). Furthermore, tumors lacking KMT2D present a blunt response to vitamin C in contrast to control group (Fig. 4C and D) with a mild reduction in tumor weight (Fig. 4E). Immunoblot analysis showed that tumors receiving vitamin C display higher expression of KMT2D in control group (Fig. 4F). Collectively, KMT2D functions as the key target of TET2 to mediate an antitumor effect of vitamin C.

### Deficiency of KMT2D sensitizes HCC to cisplatin

**Fig. 5.**
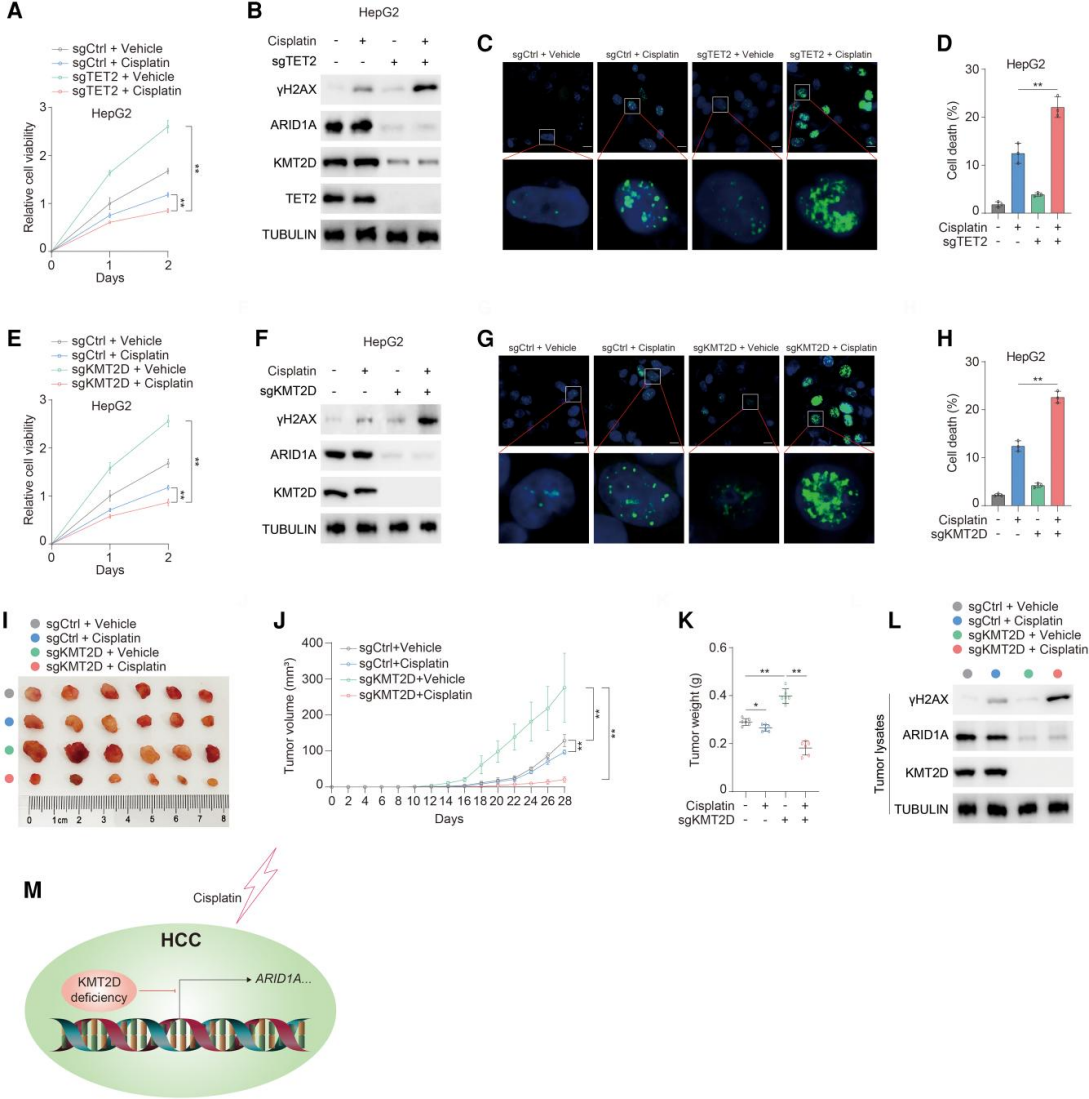
Deficiency of KMT2D sensitizes HCC tumor to cisplatin. A, B) sgCtrl and sgTET2 HepG2 cells treated with or without 10 μM cisplatin for the indicated days. Cell viability (A) and indicated proteins (B) were analyzed. Immunoblotting analysis was performed using the indicated antibodies (B). C) sgCtrl and sgTET2 HepG2 cells treated with or without 20 μM cisplatin for 6 h. γH2AX level was determined using immunofluorescence. Scale bar, 10 μm. D) sgCtrl and sgTET2 HepG2 cells treated with or without 20 μM cisplatin for 24 h. Cell death was quantified by propidium iodide staining. E, F) sgCtrl and sgKMT2D HepG2 cells treated with or without 10 μM cisplatin for the indicated days. Cell viability (E) and indicated proteins (F) were analyzed. Immunoblotting analysis was performed using the indicated antibodies. G) sgCtrl and sgKMT2D HepG2 cells treated with or without 20 μM cisplatin for 6 h. γH2AX level was determined using immunofluorescence. Scale bar, 10 μm. H) sgCtrl and sgKMT2D HepG2 cells treated with or without 20 μM cisplatin for 24 h. Cell death was quantified by propidium iodide staining. I–L) sgCtrl and sgKMT2D MHCC97H cells were subcutaneously injected into athymic nude mice administrated with or without cisplatin. Representative tumor xenografts (I). Mice were sacrificed in week 4, and tumor volume (J) and weight (K) were calculated. *n* = 6 independent animals. Tumor lysates harvested were subjected to immunoblotting analysis as indicated. Immunoblotting analysis was performed using the indicated antibodies (L). M) Schematic of KMT2D-mediated sensitivity of HCC to cisplatin. Data are presented as mean ± SD, *n* = 3 independent repeats. Unpaired, two-tailed t test; *P < 0.05, **P < 0.01.

Given that ARID1A sustains cell proliferation in response to DNA damage and cancer cells lacking ARID1A are potentially vulnerable to cisplatin (27), we next tried to unravel potential clinical significance of KMT2D in management of HCC. Compared with control group, TET2 knockout HCC cells are prone to proliferation arrest (Figs. 5A and S5A) in response to cisplatin with higher expression of γH2AX (Figs. 5B and S5B), γH2AX foci (Fig. 5C), and cell death (Figs. 5D and S5C). Likewise, deficiency of KMT2D sensitizes HCC cells to cisplatin with severe arrest of cell viability (Figs. 5E and S5D), elevated expression of γH2AX (Figs. 5F and S5E), γH2AX foci (Fig. 5G), and cell death (Figs. 5H and S5F). To further verify the above findings in vivo, we therefore subcutaneously injected control and KMT2D knockout HCC cells into athymic nude mice. Tumors lacking KMT2D present growth advantage over control group (Fig. 5I and J). But KMT2D-deficient tumors are susceptible to cisplatin treatment with dramatic growth arrest (Fig. 5I and J), reduced tumor weight (Fig. 5K), and elevated expression of γH2AX (Fig. 5L) in contrast to control group. Together, deficiency of KMT2D promotes tumor growth and sensitizes HCC to cisplatin (Fig. 5M).

### TET2–KMT2D axis correlates with prognosis of LIHC

**Fig. 6.**
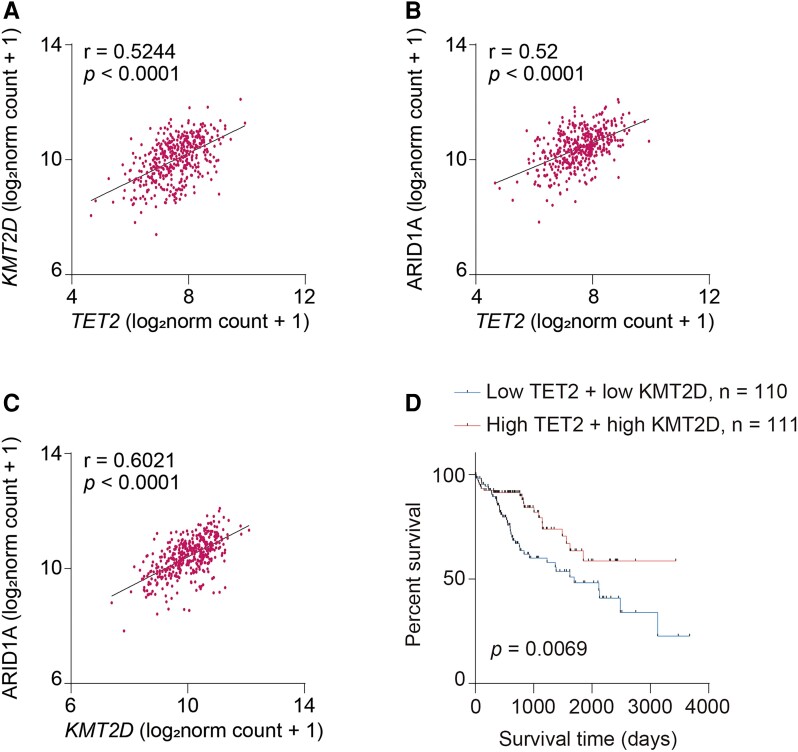
TET2–KMT2D axis correlates with prognosis of HCC. A–C) Pearson’s correlation of *TET2* and *KMT2D* (A), *TET2* and *ARID1A* (B), or *KMT2D* and *ARID1A* (C) in LIHC of TCGA database. The datasets show the gene-level transcription estimates, as in log_2_(*x* + 1)-transformed RSEM normalized count. D) TET2–KMT2D axis harbors potential significance for prognosis of LIHC. The combination of TET2 and KMT2D expression is a potential marker for prognosis of LIHC. LIHC samples from TCGA database were divided based on the expression of TET2 and KMT2D and the prognosis of these groups was analyzed.

The clinical relevance of TET2–KMT2D–ARID1A axis in LIHC was further validated by analysis of TCGA database. In line with our in vitro study, either the level of *KMT2D* or *ARID1A* presents a positive relationship with the amount of *TET2* in patients with LIHC (Fig. 6A and B). Accordantly, the expression of *ARID1A* is positively related to *KMT2D* as well (Fig. 6C). Moreover, patients with high expression of *TET2* and *KMT2D* present a better prognostic outcome in contrast to patients with low expression of the counterparts (Fig. 6D), implying that combination analysis of TET2 and KMT2D harbors clinical potential for patients with HCC. These results underline the vital role of TET2–KMT2D axis in prognosis of HCC.

## Discussion

Our findings demonstrated that KMT2D and ARID1A are per se targets of TET2 in HCC cells. KMT2D recruits TET2 to enable self-transcription and expression of ARID1A via oxidation of 5mC in promoters. Moreover, KMT2D exerts a tumor-suppressive role and acts as a bridge in mediating the antitumor effect of vitamin C in HCC. Deficiency of KMT2D sensitizes HCC tumors to cisplatin with elevated degree of DNA damage. Furthermore, TET2–KMT2D axis correlates with prognosis of HCC. High expression of *TET2* and *KMT2D* presents a better prognostic outcome of patients with HCC. These results not only reveal the cross-talk between TET2 and KMT2D and underscore the physiological role of KMT2D in HCC, but also shed light on the potential clinical relevance of TET2–KMT2D axis.

*KMT2D* (also known as *MLL2* in humans and *Mll4* in mice) belongs to the KMT2 family, which is highly conserved throughout eukaryotes and promotes genome accessibility and transcription via coordinating methylation of histone H3 lysine 4 (28). KMT2D functions as a bona fide tumor suppressor across cancers (21, 29, 30). Deficiency of KMT2D drives sensitivity of lung squamous cell carcinoma to RTK–RAS inhibition (31). Moreover, KMT2D was identified as a major modulator of immune checkpoint blockade (ICB). Lack of KMT2D sensitizes tumors to ICB via augmenting tumor immunogenicity (32). Given the prevalence of mutations across cancers, exploration on the molecular behavior of KMT2D may bring broad implications for patient stratification.

The notion that utilization of vitamin C for cancer therapy has experienced a controversial history due to lack of distinct exploration of molecular mechanism. Recently, vitamin C was identified as an agonist of TET2 by acting as a cofactor of TET2 as well as an electron donor to generate Fe^2+^ from Fe^3+^ (33). Besides TET2-dependent epigenetic reprogramming, vitamin C can also target other two vulnerabilities of cancers, that is, redox balance and oxygen sensing. Except for iron redox balance, pharmacological vitamin C produces excess extracellular H_2_O_2_, which contributes to DNA damage and finally leads to toxicity to tumors (34). Moreover, various solid tumors developed active HIF1 to adapt hypoxic microenvironment, since HIF1 functions as a master transcription factor to initiate transcription of a wide range of genes to maintain tumor growth (35). Similar to TET2, vitamin C contributes to HIF1 hydroxylase activity by recycling Fe^2+^. Therefore, vitamin C treatment could impair HIF1α activity and suppress tumor growth by enhancing HIF1 hydroxylase activity (36). Considering that vitamin C has been utilized for a clinical trial (33), our study not only provokes clear biomarkers and molecular mechanism on the basis of TET2–KMT2D axis, but also proposes an alternative strategy for management of HCC.

Over the past few decades, tremendous progress in antitumor drug discovery has been made as well as continuous improvements of available therapeutic options and strategies. But drug resistance remains the major challenge in management of cancers and threatens human health. Since epigenetic aberrations become hallmark of cancers, epi-drugs, drugs targeting epigenetic modulators, were gradually developed as yet. Nevertheless, the efficacy of epi-drugs is mainly confined to hematological malignancy (37), highlighting the need for a far more effective route for utilization of epi-drugs in solid tumor. Our findings suggested that synergistic combination of chemotherapy and epi-drugs targeting KMT2D may provide an alternative option for HCC therapy.

## Methods

### Cell culture

Cells were maintained in DMEM (Meilunbio) supplemented with 10% FBS (Biological Industries).

### Animal study

MHCC97H cells were washed twice with PBS and concentrated to 10^6^ per 100 μL in PBS. 100 μL of cells was subcutaneously injected into right back flank of 6-week-old male BALB/c nude mice. Vitamin C treatment was performed by intraperitoneal injection of 2 g/kg bodyweight daily starting at day 7 after transplantation. Cisplatin treatment was performed by intraperitoneal injection of 4 mg/kg bodyweight twice a week for 3 weeks at day 7 after transplantation. Tumor volume was calculated as volume = width^2^ × length × 0.5. All animal experiments were approved by the Institutional Animal Care and Use Committee of Renji Hospital, Shanghai Jiao Tong University School of Medicine. Tumor size must not exceed 20 mm at the largest diameter in an adult mouse, and no experiments in this study generated a tumor burden over this limit.

### Antibodies

Antibody that recognizes KMT2D (HPA035977) was purchased from Sigma. Antibodies that recognize TET2 (18950), Histone H3 (4499S), H3K4me1 (5326S), H3K4me2 (4658S), and H3K4me3 (9751S) were purchased from CST. Antibody that recognizes TET2 (ab243323) for immunofluorescence analysis was purchased from Abcam. Antibody that recognizes γH2AX (ET1602-2) was purchased from HUABIO. Antibody that recognizes ARID1A (A18650) was purchased from ABclonal. Antibody that recognizes TUBULIN (11224-1-AP) was purchased from Proteintech.

### Materials

Vitamin C (MB2693) was purchased from Meilunbio. Cisplatin (T1564), protease inhibitor cocktail (C0001), and phosphatase inhibitor cocktail (C0002) were purchased from TargeMol. EZ Protein any KD PAGE kit (AP15L055) was purchased from Life-iLab. ChIP assay kit (56383) was purchased from CST. 5mC and 5hmC ChIP assay kit (55009 and 55010) was purchased from Active Motif.

### sgRNAs and shRNAs construction

The sgRNAs were generated by annealed oligonucleotides and cloned into pLentiCRISPRv2. The target sequence 1 of *TET2* was 5′-GATTCCGCTTGGTGAAAACG-3′; the target sequence 2 of *TET2* was 5′-TACCGTTCAGAGCTGCCACC-3′; the target sequence 1 of *KMT2D* was 5′- CGTTGTGCTCTCTGTAACTG-3′; the target sequence 2 of *KMT2D* was 5′- AACCGACGGAGGGCGTAGTG-3′; the target sequence 1 of *ARID1A* was 5′- CAGCAGAACTCTCACGACCA-3′; the target sequence 2 of *ARID1A* was 5′- TGAGCGAGACTGAGCAACAC-3′. The shRNAs were generated by annealed oligonucleotides and cloned into pLKO.1. The target sequence 1 of *KMT2D* was 5′- CCCACCTGAATCATCACCTTT-3′; the target sequence 2 of *KMT2D* was 5′- CCTCGCCTCAAGAAATGGAAA-3′. The target sequence of *TET2* was 5′- GGGTAAGCCAAGAAAGAAA-3′.

### Plasmid and protein expression

Genes encoding TET2 FL, N1127, and CD were cloned into p3-Flag vector and transfected into cells transiently. Genes encoding KMT2D PHD1-3, PHD4-6, and PHD7+SET were cloned into pLVX-HA vector and transfected into cells stably via lentivirus infection after selection using puromycin. For preparation of lentivirus, 6 μg overexpression, sgRNAs or shRNAs plasmids, 4.5 μg psPAX2, and 1.5 μg pMD2.G were transfected into HEK-293T cells in 100-mm dishes. All plasmids were transfected using EZ Trans (Life-iLab) according to the manufacturer's instructions. For purification of GST-PHD6, gene encoding PHD6 was cloned into pGEX-4T-1. GST-PHD6 domain was purified from *Escherichia coli* using GST beads. For purification of TET2-CD domain, 293T cells were transfected with 10 μg Flag-TET2 CD plasmid transiently. TET2-CD domain was immunoprecipitated using Flag beads and purified with Flag peptides.

### Cell viability assay

5 × 10^3^ cells per well were seeded in 96-well plates for assay. Cells were incubated with 10% CCK8 reagents (New Cell & Molecular Biotech) that was diluted in DMEM at 37 °C for 1–4 h until the visual color conversion occurred. Cells were then analyzed by measuring the absorbance at 450 nm using a microplate reader.

### Cell death detection

Cell death was quantified by propidium iodide staining (Meilunbio). Briefly, cells were seeded into 12-well plates at a density of 50% confluence. After treatment with 20 μM cisplatin for 24 h, cells including floating dead cells were collected and stained with propidium iodide. The percentage of the propidium iodide-positive dead cell population was analyzed using flow cytometry.

### Gene expression and RNA-seq analysis

Total RNA was isolated using EZ-press RNA purification kit (EZ Bioscience) following the manufacturer's instructions. We synthesized cDNA from 1 μg total RNA using EZ Bioscience-RT mix (EZ Bioscience). The cDNA in triplicate was assessed for target mRNA levels by quantitative real-time PCR with SYBR qPCR Master Mix (Yeasen). We calculated relative mRNA levels normalized to human *TUBULIN* levels in the same samples. The qPCR primer sequences were as follows: *TUBULIN*: 5′-TCGATATTGAGCGTCCAACCT-3′ (forward) and 5′-CAAAGGCACGTTTGGCATACA-3′ (reverse); *KMT2D*: 5′-GAGCTACGGCGCTTTGAGTT-3′ (forward) and 5′-AGGGAAACCAATCTGTGATAGGT-3′ (reverse); *ARID1A*: 5′-CCTGAAGAACTCGAACGGGAA-3′ (forward) and 5′-TCCGCCATGTTGTTGGTGG-3′ (reverse). RNA-seq analysis was performed by Origin-gene (Shanghai, China).

### Whole-genome bisulfite sequencing

Whole-genome bisulfite sequencing and data analysis were conducted by AceGen.

### Western blot and immunofluorescence staining

For western blot sample preparation, cells or tissue samples were collected with 0.5% NP-40 lysis buffer containing 1× protease inhibitor cocktail, followed by mixing with 5× SDS-loading buffer (Yeasen).

For immunofluorescence staining, HepG2 cells were seeded onto coverslips at 50% confluency for 1 day prior to experiment. After rinsing with cold PBS, cells were fixed with 4% paraformaldehyde (Beyotime) for 15 min. The coverslips were incubated with 0.3% Triton X-100 in PBS at room temperature for 15 min, followed by washing with PBS. Cells on coverslips were blocked with goat serum (Beyotime) at room temperature for 30 min. The slides were incubated with primary antibodies in QuickBlock Primary Antibody Dilution Buffer (Beyotime) at 4 °C overnight. After washing with PBS, the slides were incubated with secondary antibodies (Alexa488- or Alexa555-conjugated) in QuickBlock Secondary Antibody Dilution Buffer (Beyotime) at room temperature for 30 min. The slides were mounted with antifade reagent, and samples were observed using an immunofluorescence microscope (Nikon).

### Docking analysis

Docking analysis was performed via GRAMM server (https://gramm.compbio.ku.edu). The structures of TET2 CD and KMT2D PHD6 were downloaded from the PDB with accession numbers 4NM6 and 8U2Y, respectively.

### PD assay

PD assay was carried out by incubating 5 μg GST-PDH6 and 5 μg TET2 CD at 4 ℃ overnight and purifying using GST beads. The interaction was examined by Coomassie brilliant blue staining. Empty vector expressing GST tag was used as a negative control.

### ChIP assay

ChIP assay was performed according to the manufacturer's instructions. Briefly, 5 × 10^7^ cells per group were collected for assay. Cells were fixed with formaldehyde, and chromatin was fragmented by sonication. Antibodies against TET2 (1:15 dilution), KMT2D (1:15 dilution), H3K4me1 (1:50 dilution), H3K4me2 (1:50 dilution), H3K4me3 (1:50 dilution), 5mC (1:50 dilution), and 5hmC (1:50 dilution) were used for IP. Quantitative real-time PCR was used to measure the amount of bound DNA, and the value of enrichment was calculated normalized to input. Primers covering the TET2- and KMT2D-binding site of the *KMT2D* and *ARID1A* gene promoter region were used for quantitative real-time PCR. The following promoter-specific primers were used: *KMT2D* promoter region: 5′-GCAAGGGTATGGAATTAGACAG-3′ (forward) and 5′-CACACGCTGCGGGATCCTTC-3′ (reverse); *ARID1A* promoter region: 5′-GACAGACCTGGATAGGGACGC-3′ (forward) and 5′-CACGAGGCTCAGCACTGCCAT-3′ (reverse).

### TCGA data analysis

Mutual exclusivity analysis of *IDH1*, *IDH2*, *TET2*, and *KMT2D* in LIHC and AML was performed at http://www.cbioportal.org. TCGA data of LIHC were downloaded via UCSC XENA platform (https://xena.ucsc.edu/). The datasets show the gene-level transcription estimates, as in log_2_(*x* + 1)-transformed RSEM normalized count. Pearson’s correlation coefficients were calculated to analyze the relation between *TET2* and *KMT2D*, *TET2* and *ARID1A*, or *KMT2D* and *ARID1A* mRNA levels.

### Statistics and reproducibility

Statistical testing was performed using the unpaired, two-tailed Student's t test. All experiments were performed at least three times unless otherwise indicated, and representative results are shown in the figures. Figure legends indicate the *N* numbers. Analyses were performed with GraphPad Prism. Data are presented as mean ± SD *P* values < 0.05 were considered statistically significant (*P < 0.05, **P < 0.01). NS represents not significant.

## Supplementary Material

pgae504_Supplementary_Data

## Data Availability

RNA-sequencing and whole-genome bisulfite sequencing datasets are available at the NCBI SRA database under accession numbers PRJNA781771 and PRJNA782072, respectively.
