# Supplementary material for: Deficiency of TET2-mediated KMT2D self-transcription confers a targetable vulnerability in hepatocellular carcinoma
Source: PNAS Nexus. 2024 Nov 11;3(11):pgae504. doi: 10.1093/pnasnexus/pgae504 (PMC11574621; doi:10.1093/pnasnexus/pgae504)
Supplement: pgae504_Supplementary_Data [file pgae504_supplementary_data.zip › PNASNEXUS-PNASNEXUS-2024-00569RR-s01.docx]

**Figure legends**

**Fig. S1 TET2 promotes expression of *KMT2D* and *ARID1A* in HCC cells.** (**A, B**) HCC cells as indicated were transfected with or without sgRNAs targeting TET2 (sgTET2). mRNA (A) and protein (B) levels of KMT2D and ARID1A were analyzed. Immunoblotting analysis were performed using the indicated antibodies (B). (**C, D**) HCC cells as indicated were transfected with or without sgRNAs targeting KMT2D (sgKMT2D) and overexpressed with or without TET2. mRNA (C) and protein (D) levels of ARID1A were analyzed. Immunoblotting analysis were performed using the indicated antibodies (D). (**E, F**) HCC cells as indicated were transfected with or without sgRNAs targeting ARID1A (sgARID1A) and overexpressed with or without TET2. mRNA (E) and protein (F) levels of KMT2D were analyzed. Immunoblotting analysis were performed using the indicated antibodies (F). Data are presented as mean ± s.d., n = 3 independent repeats. Unpaired, two-tailed *t*-test; ** *p* < 0.01. NS, not significant.

**Fig. S2 TET2 initiates transcription of *KMT2D* and *ARID1A* via oxidizing 5mC of promoters.** (**A, B, C**) sgCtrl and sgTET2 HCC cells as indicated were transfected with or without wild-type TET2 (WT) and TET2 catalytic mutant (R1896S). 5hmC dot blot (A), RNA (B) and protein (C) levels of KMT2D and ARID1A were analyzed. Immunoblotting analysis were performed using the indicated antibodies (C). (**D, E**) sgCtrl and sgTET2 HCC cells as indicated were treated with or without 1 mM Vc for 24 h. mRNA (D) and protein (E) levels of KMT2D and ARID1A were analyzed. Immunoblotting analysis were performed using the indicated antibodies (E). (**F, G**) sgCtrl and sgKMT2D HCC cells as indicated were treated with or without 1 mM Vc for 24 h. mRNA (F) and protein (G) levels of ARID1A were analyzed. Immunoblotting analysis were performed using the indicated antibodies (G). (**H**) ChIP analysis was performed in sgCtrl and sgTET2 HCC cells as indicated using antibody against TET2. DNA enrichment was examined by quantitative real-time PCR. The y axis shows the value normalized to input. (**I, J**) ChIP analysis was performed in shTET2 HCC cells as indicated using antibody against TET2. Immunoblotting analysis were performed using the indicated antibodies (I). DNA enrichment was examined by quantitative real-time PCR. The y axis shows the value normalized to input (J). (**K, L**) ChIP analysis was performed in HCC cells overexpressing TET2 as indicated using antibody against TET2. Immunoblotting analysis were performed using the indicated antibodies (K). DNA enrichment was examined by quantitative real-time PCR. The y axis shows the value normalized to input (L). (**M, N**) ChIP analysis was performed in sgCtrl and sgTET2 HCC cells as indicated using antibodies against 5mC (M), and 5hmC (N). DNA enrichment was examined by quantitative real-time PCR. The y axis shows the value normalized to input. (**O**) Methylation level of *KMT2D* and *ARID1A* promoters were measured using whole-genome bisulfite sequencing analysis in sgCtrl and sgTET2 HepG2 cells. Data are presented as mean ± s.d., n = 3 independent repeats. Unpaired, two-tailed *t*-test; * *p* < 0.05, ** *p* < 0.01. NS, not significant.

**Fig. S3 TET2 is recruited by KMT2D to facilitates self-transcription.** (**A**) Mutations in IDH1/2-TET2 axis and KMT2D display mutual exclusivity in AML. (**B**) TET2 interacts with KMT2D. Whole cellular extracts were subjected to immunoprecipitation (IP) using the indicated antibodies in HCC cells as indicated. (**C**) ChIP analysis was performed in sgCtrl and sgKMT2D HCC cells using antibodies against TET2. DNA enrichment was examined by quantitative real-time PCR. The y axis shows the value normalized to input. (**D, E, F, G**) sgCtrl and sgKMT2D HCC cells transfected with or without WT and R1896S TET2. Immunoblotting analysis were performed using the indicated antibodies (D). Dot blot were performed using antibodies against 5hmC (E). ChIP analysis was performed using antibodies against 5mC (F), and 5hmC (G). DNA enrichment was examined by quantitative real-time PCR. The y axis shows the value normalized to input. (**H**) ChIP analysis was performed in HCC cells using antibodies against KMT2D. DNA enrichment was examined by quantitative real-time PCR. The y axis shows the value normalized to input. (**I, J, K, L**) HCC cells as indicated were transfected with or without shRNAs targeting KMT2D (shKMT2D). Protein (I) and mRNA (J) levels of KMT2D and ARID1A were analyzed. Immunoblotting analysis were performed using the indicated antibodies (I). ChIP analysis was performed in HCC cells to measure enrichment in *KMT2D* (K) and *ARID1A* (L) promoters using antibodies against H3K4me1, H3K4me2 and H3K4me3. DNA enrichment was examined by quantitative real-time PCR. The y axis shows the value normalized to input. (**M, N**) H3K4me3 enrichment in *KMT2D* (K) and *ARID1A* (L) genes of TET2 knockdown (RNAi TET2) 293T cells (left panel) and bone marrow of Tet2 knockout (Tet2 KO) mice (right panel) were analyzed using NCBI database (accession number GSE36620). Differential enrichments were marked with green box. Data are presented as mean ± s.d., n = 3 independent repeats. Unpaired, two-tailed *t*-test; ** *p* < 0.01. NS, not significant.

**Fig. S4 KMT2D is crucial for antitumor effect of vitamin C in HCC cells.** (**A**) Cell viability was analyzed in sgCtrl and sgTET2 HCC cells as indicated treated with or without 1 mM Vc for indicated days. (**B**) Cell viability was analyzed in sgCtrl and sgKMT2D HCC cells as indicated cells treated with or without 1 mM Vc for indicated days. Data are presented as mean ± s.d., n = 3 independent repeats. Unpaired, two-tailed *t*-test; * *p* < 0.05, ** *p* < 0.01.

**Fig. S5 Deficiency of KMT2D sensitizes HCC cells to cisplatin.** (**A, B**) sgCtrl and sgTET2 HCC cells as indicated were treated with or without 10 μM cisplatin for indicated days. Cell viability (A) and indicated proteins (B) were analyzed. Immunoblotting analysis were performed using the indicated antibodies (B). (**C**) sgCtrl and sgTET2 HepG2 cells treated with or without 20 μM cisplatin for 24 h. Cell death were quantified by propidium iodide staining. (**D, E**) sgCtrl and sgKMT2D HCC cells as indicated were treated with or without 10 μM cisplatin for indicated days. Cell viability (D) and indicated proteins (E) were analyzed. Immunoblotting analysis were performed using the indicated antibodies (E). (**F**) sgCtrl and sgKMT2D HepG2 cells treated with or without 20 μM cisplatin for 24 h. Cell death were quantified by propidium iodide staining. Data are presented as mean ± s.d., n = 3 independent repeats. Unpaired, two-tailed *t*-test; * *p* < 0.05, ** *p* < 0.01.
