# Supplementary figures and images for: Deficiency of TET2-mediated KMT2D self-transcription confers a targetable vulnerability in hepatocellular carcinoma
Source: PNAS Nexus. 2024 Nov 11;3(11):pgae504. doi: 10.1093/pnasnexus/pgae504 (PMC11574621; doi:10.1093/pnasnexus/pgae504)

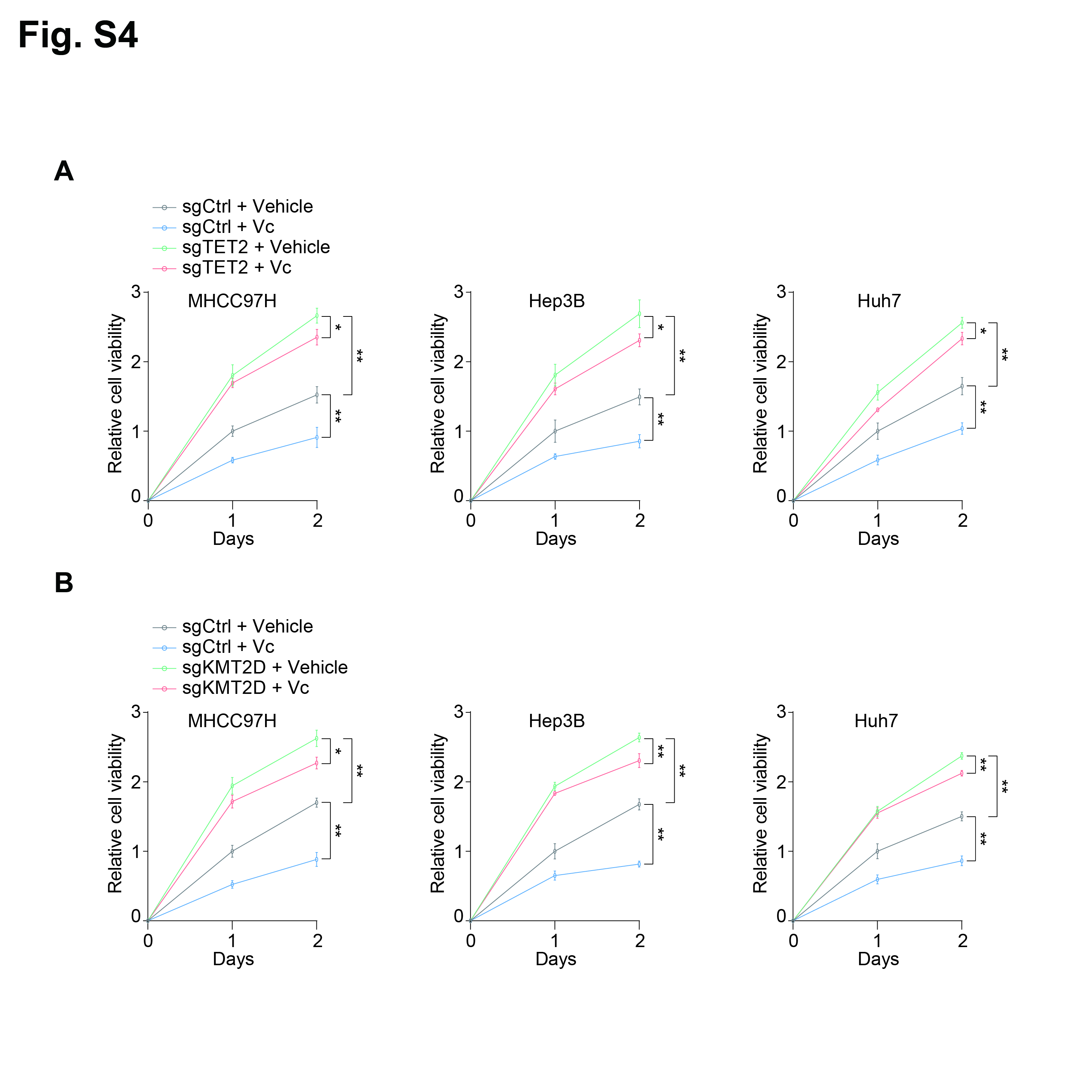

Supplement: pgae504_Supplementary_Data [file pgae504_supplementary_data.zip › PNASNEXUS-PNASNEXUS-2024-00569RR-s05.tif]

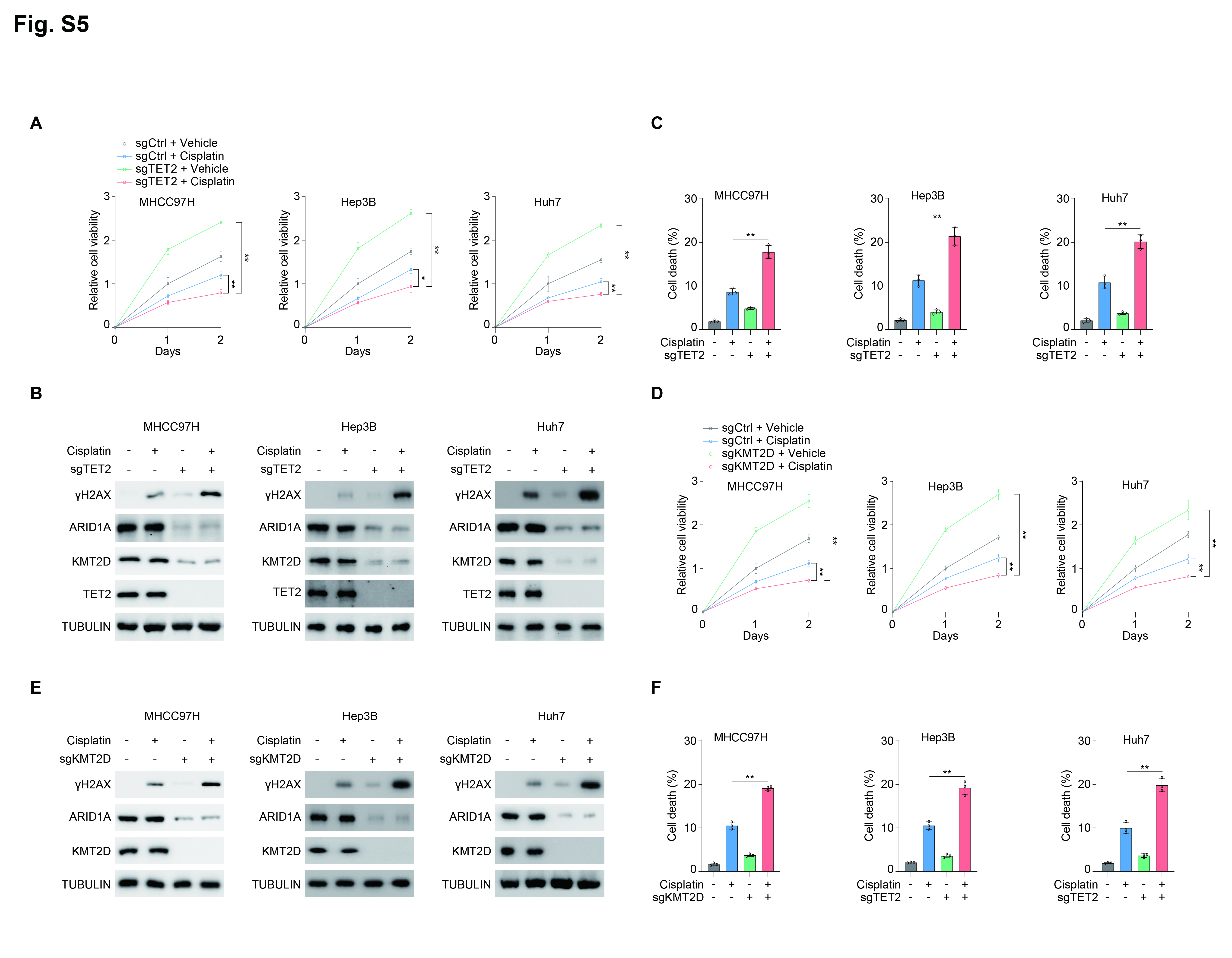

Supplement: pgae504_Supplementary_Data [file pgae504_supplementary_data.zip › PNASNEXUS-PNASNEXUS-2024-00569RR-s06.tif]
